# Analysis of online information about success rates in fertility clinics in Spain. An urgent call for action

**DOI:** 10.12688/openreseurope.20090.2

**Published:** 2025-10-22

**Authors:** Anna De Bayas Sanchez, Virginie Rozée, Manon Vialle, Kristien Hens, Joke Stryuf, Willem Ombelet, María Rosa Tapia Sánchez, Marta María Albert Márquez, Juana Farfán Montero, José Miguel Carrasco, Michaela Fuller, Vera Dimitrievska, Jana Melosca, Nathaniel Barrett, Francisco Güell Pelayo

**Affiliations:** 1 Institute for Medical Humanities, First Medical Faculty, Charles University, Prague, Czech Republic; 2 Medistella, Mediversvm s.r.o., Czech Republic; 3 INED, Research Unit “Sexual and reproductive Health and Rights", Aubervilliers, France; 4 Department of Philosophy, University of Antwerpen, Antwerpen, Belgium; 5 The Walking Egg Non-Profit Organization, Genk, Belgium; 6 Faculty of Medicine and Life Sciences, University of Hasselt, LCRC, Diepenbeek, Belgium; 7 University Rey Juan Carlos, Madrid, Spain; 8 APLICA Investigación y Traslación Cooperative, Madrid, Spain; 9 University of American College, Skopje, North Macedonia; 10 Healthgrouper, North Macedonia, North Macedonia; 11 Institute for Culture and Society, University of Navarra, Navarra, Spain; 12 Facultad de Bioética, Universidad Anáhuac México, Ciudad de México, Mexico

**Keywords:** ethics, bioethics, IVF advertising, assisted reproduction, success rates, fertility clinics, patient information

## Abstract

**Background:**

The medically assisted reproduction (MAR) industry is a booming sector of the economy, and Spain is proving to be a notable example of this industry's rise. Marketing strategies in the healthcare sector are shaped by the constant tension between providing clear, complete and appropriate information about treatments and providing information to attract and retain customers.

**Methods:**

We conducted a systematic analysis of success rate information published on the websites of Spain’s five with the strongest online presence MAR clinics as of November 2023. The analysis examined terminology, certifications and audits by external bodies, references to data sources, age-specific success rates, and generalised claims about treatment effectiveness.

**Results:**

We identified significant deficiencies and ambiguities in how clinics present success rate data. Common issues included vague definitions, lack of certification details, absence of reliable data sources, and failure to differentiate outcomes by age or treatment type. Several clinics used overly optimistic or potentially misleading statistics, making it difficult for patients to assess their realistic chances of success. We provide recommendations aimed at improving consumer protection and regulating advertising practices. We also emphasise that presenting clear and truthful success rate information is not only a legal requirement but also an ethical duty in reproductive healthcare.

**Conclusions:**

Stronger regulation of success rate reporting in Spain’s fertility sector is urgently needed. We recommend that external authorities oversee and standardise data reporting to ensure transparency and prevent misleading practices. A centralised public body should be responsible for monitoring, collecting, and managing this data to protect patient rights and improve trust in fertility care.

## Introduction

The medically assisted reproduction (MAR) industry is a rapidly expanding sector of the economy, driven by rising global infertility rates, which affect approximately 48 million couples and 186 million individuals worldwide. More than 10 million children have been born using MAR techniques ( WHO, 2021), and the demand for these services continues to increase, now accounting for up to 7.9% of all births in Europe ( Wyns *et al.*, 2022). On a global scale, Spain is one of the leading countries in terms of the number of clinics and total MAR cycles performed and is one of the main European destinations for reproductive tourism ( Lafuente-Funes *et al.*, 2023). According to the Spanish Fertility Society (SEF) registry, 165,453 IVF cycles and 33,818 artificial inseminations were performed in 2021, representing an 11.7% increase compared to 2019 ( SEF, 2021). The MAR industry has experienced significant growth, with the global in vitro fertilisation market reaching a valuation of USD 25 billion in 2022. Forecasts suggest that the market will exceed USD 59.45 billion by 2032. In Europe, the MAR market reached USD 7.75 billion in 2022 and is predicted to be worth over USD 13.05 billion by 2032, as a result of increasing medical tourism and legal reforms related to MAR techniques ( Precedence Research, 2023). The internet has transformed how patients access MAR information. While patients once relied on doctors, they now have the ability to conduct online research on clinics in advance ( Zhu *et al.*, 2024). However, this shift has not eliminated the information gap between patients and providers, often leading to marketing strategies that prioritise client acquisition over transparent communication of clinical outcomes. Major (2019) emphasises that patients typically possess less information than providers, including knowledge about alternative treatments and their effectiveness.

Many fertility clinics present success rates using a wide range of inconsistent measures, making it difficult for patients to make informed choices ( Wilkinson *et al.*, 2017). The efficiency of the treatment in achieving its intended outcome is a central consideration. In the case of fertility treatment, the objective is the birth of a healthy child. Success in MAR should be understood as a multidimensional concept that includes not only live birth rates but also patient satisfaction, cost-effectiveness, and time to pregnancy ( Rienzi *et al.*, 2021). A woman’s age is the most important prognostic factor in fertility treatment. While the chances of becoming pregnant remain relatively stable up to the age of 30, they begin to decline significantly from age 37 onwards. For this reason, it is critical that information about success rates be contextualised by age. Treatment uptake data confirm this trend: according to the Spanish Fertility Society (SEF), around 50% of women attending clinics are over 40 years old, and approximately 75% of all cycles and transfers involving egg donation are performed for women over 40. One of the most critical factors for informed decision-making in fertility treatment is the success rate associated with each technique. This information should be presented with maximum clarity and accuracy to allow patients to make well-informed choices.

These issues should be read in the light of Spain’s regulatory context: MAR clinics must be authorised by the regional health authorities and report activity and outcomes to the Spanish Fertility Society (SEF). However, there is no centralised system for external auditing or mandatory certification of the data published on clinic websites.

In this paper, we present a systematic analysis of the information about success rates available on the websites of five of the most representative fertility clinics in Spain. To our knowledge, this is the first systematic content analysis of Spanish fertility clinic websites focused specifically on how success rate data is communicated to potential patients.

## Methods

Patient and Public Involvement: Although "Fertility Europe", the largest fertility patient association in Europe, was not a member of the scientific team of the project in which this research is framed, the association took part in the B2-InF consortium meetings where the checklist for the analysis of clinic websites was discussed and refined with their input.

To identify MAR clinics with the strongest online presence in Spain, we conducted internet searches using the five most common Google search keywords related to fertility treatment: ‘fertilisation’,‘insemination‘, ‘infertility’, ‘pregnancy IVF’ (in vitro fertilisation), and ‘ICSI’ (intracytoplasmic sperm injection). This method was intended to capture the clinics most likely to be encountered by patients online, based on visibility and accessibility of information. Search results for each keyword were selected in order of appearance, excluding magazine articles, blogs, and clinics without their own laboratory. The selected clinics were Ginefiv, Instituto Bernabeu, Institut Marqués, FIV Valencia, and IVI-RMA. The selection process took place between July and August 2021. All chosen clinics operate in the private sector, which provides the majority of fertility treatments in Spain. These clinics play a significant role in shaping public perceptions of MAR, particularly regarding effectiveness, accessibility, and treatment outcomes.

Our focus was on the patient perspective: patients typically rely on online searches rather than scientific registries when seeking fertility care. Therefore, clinics most visible on Google are the most influential in shaping patient decisions. While this approach does not capture the full universe of Spanish clinics, it provides relevant insights into the information that patients are most likely to encounter.

Once selected, the websites of these five clinics were thoroughly examined to determine the information available in the local language. Data collection followed a systematic approach using specially designed templates categorised by theme, incorporating both textual and graphical content. Success rates were among the key themes analysed. The collected information was analysed in July and August 2023, and the clinic websites were reviewed again in March 2025 to verify any changes. The analysis focused on the terminology used to define treatment success rates (live birth, clinical pregnancy, or biochemical pregnancy), differentiation of success rates by age and technique (artificial insemination, IVF/ICSI, with or without gamete donation), and generalised quantitative descriptions of treatment effectiveness, and certification, and auditing by external bodies, reference to data sources.

This research has been conducted under the European Project “B2-InF: Be Better Informed About Fertility”. The B2-InF Project was approved by the Research Ethics Committee of the University of Navarra (Project No. 2021.004, approved 29/01/2021). Data and materials related to this research are publicly available in the B2-InF project repository on Zenodo ( Carrasco *et al.*, 2023).

## Results

### Criteria, clarification and definition of success rate

All clinics included in our analysis report success rates as cumulative per cycle, including outcomes from both fresh and frozen embryo transfers within the same treatment cycle.

Table 1 shows the criteria used to define success rates on the websites of the clinics surveyed. In particular, we analysed whether the success rate is defined as live birth, clinical pregnancy, or biochemical pregnancy, and whether these criteria are explicitly stated and explained.

**Table 1.** Criteria on which the success rate is based.

	Live birth	Clinical pregnancy ^a^	Biochemical ^b^	Explanation *	Definition**
Ginefiv ^1^	No	No	Yes	No	No
Instituto Bernabeu ^2^	No	Yes	Yes	No	No
Institut Marquès ^3^	Yes	Yes	Yes	Clinical pregnancy/ “Born”	No
FIV Valencia ^4^	No	No	Yes	No	No
IVI-RMA ^5^	No	No	Yes	biochemical pregnancy tests	Yes

*^a^ Clinical pregnancy refers to the confirmation of the presence of a heartbeat and gestational sac by ultrasound at 6–7 weeks of pregnancy*.

*^b^ Biochemical pregnancy refers to the confirmation of the human chorionic gonadotropin (hCG) hormone in the urine, a test that indicates embryo implantation has occurred*.

* Whether “biochemical pregnancy” or “clinical pregnancy” is specified.

^1^ https://www.ginefiv.com/en/discover-ginefiv/ginefiv-success-rates/ (Consulted on March 31, 2025)

^2^ https://www.institutobernabeu.com/es/estadisticas/ (Consulted on March 31, 2025)

^3^ https://institutomarques.com/resultados/ (Consulted on March 31, 2025)

^4^ https://www.fivvalencia.com/es/fertilizacion-ovulos/icsi/ (Consulted on March 31, 2025)

^5^ https://ivi.es/preguntas-frecuentes/tasas-de-exito/ (Consulted on March 31, 2025)

• Of the five clinics surveyed, all provided information about success rates in terms of biochemical pregnancy. For three clinics, this is the only success rate information provided, while the other two also report clinical pregnancy rates.

• Only one clinic provides data on live birth rates. Additionally, only two of the clinics explicitly refer to 'biochemical pregnancy' and 'clinical pregnancy' using those terms. None of the clinics clearly explains the meaning of the terms 'biochemical pregnancy' or 'clinical pregnancy'.

### Online information on success rate and age

In the clinics surveyed (see Table 2), we analysed the information provided about success rates in relation to age. We found that all of the clinics surveyed presented success rates by age group, although these groupings vary from clinic to clinic.

**Table 2.** Success rates according to age range.

	AI	IVF/ICSI	Egg donation
Ginefiv ^6^	No range	<30, 30–34, 35–39, 40–45, >45	35–39, 40–45, >45
Instituto Bernabeu ^7^	No range	<35, 35–39, >40	No range
Institut Marques ^8^	<35, 35–39, >40	<35, 35–39, >40	No range
FIV Valencia ^9^	No range	<35, 35–39, >40	No range
IVI-RMA ^10^	No range	<29, 30–34, 35–39, 40–44	No range

*AI: Artificial Insemination* *IVF/ICSI: In Vitro Fertilisation and Intracytoplasmic Injection*.

^6^ https://www.ginefiv.com/en/discover-ginefiv/ginefiv-success-rates/ (Consulted on March 31, 2025)

^7^ https://www.institutobernabeu.com/es/estadisticas/ (Consulted on March 31, 2025)

^8^ https://institutomarques.com/resultados/ (Consulted on March 31, 2025)

^9^ https://www.fivvalencia.com/es/fertilizacion-ovulos/icsi/ (Consulted on March 31, 2025)

^10^ https://ivi.es/preguntas-frecuentes/tasas-de-exito/ (Consulted on March 31, 2025)

• Four clinics do not provide age-related success rates for donor oocytes, and only one of these also does provide age-related success rates for artificial insemination.

• Only one clinic provides information for an age range over 45.

### Success rates and MAR techniques

We also analysed the information on success rates according to the different techniques and procedures offered (see Table 3).

**Table 3.** Distinction of success rates by MAR techniques.

	IVF/ICSI	Egg donation
Ginefiv ^11^	No	Yes
Instituto Bernabeu ^12^	No	Yes
Institut Marqués ^13^	No	Yes
FIV Valencia ^14^	Yes	Yes
IVI-RMA ^15^	No	Yes

^11^ https://www.ginefiv.com/en/discover-ginefiv/ginefiv-success-rates/ (Consulted on March 31, 2025)

^12^ https://www.institutobernabeu.com/es/estadisticas/ (Consulted on March 31, 2025)

^13^ https://institutomarques.com/resultados/ (Consulted on March 31, 2025)

^14^ https://www.fivvalencia.com/es/fertilizacion-ovulos/icsi/ (Consulted on March 31, 2025)

^15^ https://ivi.es/preguntas-frecuentes/tasas-de-exito/ (Consulted on March 31, 2025)

• All of the websites consulted differentiate between success rates for patients with and without egg donation.

• Only one of the five clinics makes a distinction between the success rates for those who undergo IVF and those who undergo ICSI.

Also, because success rates vary according to the techniques used, this information needs to be clearly differentiated. We find, however, that the four clinics consulted give a combined success rate for IVF and ICSI, and it is not clear whether this is an average of the two. With regard to gamete donation, all clinics differentiate success rates based on whether donor gametes are used. However, a notable pattern is that four of the clinics reviewed present a single success rate for egg donation, regardless of the recipient's age.

### Generalised quantitative descriptions of effectiveness

For each clinic website, in addition to examining systematic presentations of success rates, we looked for other quantitative descriptions of overall success rates or expected results—for example, those found in the general narrative of the clinic (see Table 4). In four of the five clinics, we found at least one general quantitative description of success rates, i.e., quantitative information that does not specify any relation to age, technique, or the use of gamete donation.

**Table 4.** Non-specific quantitative information.

Ginefiv ^16^	*“The pregnancy rate per cycle is 75%, and the cumulative pregnancy rate after three cycles exceeds 90%.”*
Instituto Bernabeu ^17^	*“Our statistics are certified and audited: after three treatments 96% of our patients achieve pregnancy”.*
Institut Marquès ^18^	*“Thus, our success rates, audited by the government of Catalonia, are among the highest in Europe. 9 out * *of 10 patients who come to us achieve pregnancy in one or more attempts”.*
FIV Valencia ^19^	*“FIV Valencia has impressive success rates in egg donation treatments, with a cumulative pregnancy rate of * *95%, making it one of the leading clinics in Europe.“*
IVI-RMA ^20^	*“ IVI has the best pregnancy rates, 9 out of 10 couples who trust us achieve their goal of becoming parents”.*

^16^ https://www.ginefiv.com/reproduccion-asistida/ovodonacion/#:~:text=Este%20es%20un%20programa%20con,3%20ciclos%20supera%20el%2090%25 (Consulted on March 31, 2025)

^17^ https://www.institutobernabeu.com/es/estadisticas/ (Consulted on March 31, 2025)

^18^ https://institutomarques.com/reproduccion-asistida/tecnologia-avanzada/embryoscope/ (Consulted on March 31, 2025)

^19^ https://www.fivvalencia.com/es/Donacion-de-ovulos/ (Consulted on March 31, 2025)

^20^ https://ivi.es/preguntas-frecuentes/ivi-en-el-mundo/ (Consulted on March 31, 2025)

Concerning “non-specific quantitative information,” in all of the clinics consulted, we found claims regarding the high effectiveness of MAR, but without specifying techniques, procedures, or age. On three clinics' websites, the same message is repeated: 9 out of 10 achieve pregnancy. Two clinic websites present this information in connection with “achieving the expected results” and “the goal of becoming parents,” which strongly implies that 90% of patients achieve a live birth.

### Limited certification and transparency of success rate data

Only two clinics state that the data from which they derive success rates have been certified. One clinic states that its data have been certified by a relevant authority. Although access to a copy of the certificate is provided, this signed document is not sufficient for substantiation. It does not provide access to evidence-based data to corroborate the figures provided. On the other hand, it should be noted that the certificate does provide a detailed description of the process by which the certifying agency verified the data. Another clinic only claims that its data on success rates have been certified by a public body, but without providing access to this certificate or any further description of the certification process.

## Discussion

We found few studies focusing on how success rates are advertised in assisted human reproduction clinics. One such study was conducted by the Human Fertilisation and Embryology Authority (HFEA) in the United Kingdom ( Committee of Advertising Practice, 2021). After analysing the information promoted within the sector, the HFEA warned of a lack of transparency in the presentation of success rate statistics. Specifically, they expressed concern that results are often reported only for patients under 35 years of age and that the terminology used is unclear. The HFEA also recommended that percentages be presented as “live birth rate per embryo transfer.” Another study ( Sauerbrun-Cutler *et al.*, 2021) examined information provided by over 90% of U.S. clinics through their websites. According to the Society for Assisted Reproductive Technology (SART) guidelines on advertising, live birth rates per cycle start (intended retrieval) must be prominently displayed first, followed by rates per egg retrieval and per embryo transfer, with success rates reported for each SART-defined age category. Omitting live birth data per cycle start is not permitted. SART categorises maternal age as follows: <35, 35–37, 38–40, 41–42, and >42 years. The study analysed information from 361 U.S. clinics and found that only 10.5% reported success rates in terms of live births per transfer, retrieval, and cycle.

In the Spanish context, ART clinics must be authorised by the competent regional health authorities and are required to report their activity and outcome data to the Spanish Fertility Society (SEF). The SEF compiles and publishes annual reports with aggregated national data, covering a wide range of MAR techniques. However, the publication of clinic-specific success rates remains voluntary, and there is no centralised mechanism for external auditing or certification of the data published on individual clinic websites. This absence of independent oversight contributes to the lack of transparency and consistency we observed in the success rate data presented online.

Moreover, none of the clinic websites analysed disaggregated success rate data by fertilisation technique (i.e., IVF versus ICSI), making it impossible for patients to know whether reported outcomes are associated with one method or the other. This lack of transparency is particularly relevant given that, according to the 2022 SEF Registry, ICSI accounts for approximately 88.2% of initiated cycles in Spain ( Sociedad Española de Fertilidad (SEF), 2022). Patients are therefore unable to assess whether the widespread use of ICSI—often even in the absence of male factor infertility—is supported by outcomes reported at the clinic level. In fact, based on data provided by the French government, the success rate of ICSI is only slightly higher than that of IVF, and not always. Studies indicate that when the cause of infertility is not of paternal origin, the success rates of IVF are better than those of ICSI, although it is debatable which technique is more beneficial in other cases ( de Bantel-Finet *et al.*, 2022).

Given ICSI’s widespread use in Spain, greater transparency would allow patients to understand when IVF and ICSI are clinically indicated. In light of this evidence, and given the different costs associated with using one or the other, information about success rates should enable patients to take these factors into consideration.

Given the widespread expectation that fertility treatment will result in the birth of a healthy child, clinics cannot comply with consumer law unless they provide success rate information explicitly defined in terms of live births. Although some fertility clinics in Spain may have a high proportion of international patients, they represent only about 10% of all treatment cycles nationwide, according to the latest reports from the Spanish Fertility Society (SEF). The number of domestic patients alone is higher than that reported by most other countries, providing a sufficiently large and robust dataset to estimate live-birth success rates with confidence.

Faced with this ambiguity and lack of clarity, some governments (e.g., France) have set up a public website where citizens can find success rates presented exclusively in terms of birth ( Agence de la biomédecine, 2025), and have prohibited clinics from providing their own criteria and results. Similarly, Australia and New Zealand Assisted Reproduction Database ( FSANZ), Society for Assisted Reproductive Technology (SART), and the Centers for Disease Control and Prevention (CDC), have created websites that must be accessible from the websites of all clinics. On these platforms, patients can enter personal data to receive an estimate of their chance of live birth, based on national data and clinical parameters.

 In relation to this issue, it is worthwhile to point out a deficiency in the way information about success rates is managed by the Spanish Fertility Society (SEF). The SEF publishes a yearly report (“Registro Nacional de Actividad – Registro SEF”) that presents official national statistics based on data collected from all clinics, covering all the MAR techniques performed in Spain in a given year. In this document, information is presented as an average over just three age groups: <35, 35–39, and ≥40.

Although donor age is the principal determinant of success in donor-egg IVF, recent evidence shows that recipient age also significantly influences outcomes, with implantation failure and pregnancy loss risks increasing progressively after the age of 40 ( Sebastian-Leon *et al.*, 2025). This indicates that both own-egg and donor-egg cycles benefit from more precise age stratification than is currently provided in Spain.

Another dimension not addressed on clinic websites is whether success rates are reported per initiated cycle, per egg retrieval, or per embryo transfer. These yield different figures, and without clarification patients may misinterpret the likelihood of success. Providing both perspectives with clear explanations would enhance transparency.

We consider this information to be insufficient for making an informed decision about fertility treatment and indicative of a lack of involvement and oversight on the part of the Ministry of Health, which is responsible for establishing standards for data transparency in the fertility industry.

In the preceding analysis of information provided by Spanish clinics, our focus has been on the clarity and accuracy of information related to success rates. We believe that this analysis shows that the information provided does not meet national and European legal standards for advertising and publicity ( Art. 3 of the Law 34/1988; ICC Code, 2018). Potential patients should not be misled into believing that the success rate of a procedure is significantly higher than it actually is. Such statements violate Spanish and European consumer protection laws on truthful advertising, which prohibit any advertising that, through false or misleading information—or the omission of essential details—leads consumers to make misinformed decisions. Even if clinics change their information policies so that success rates are clearly and accurately presented, however, another issue needs to be confronted. According to Spanish legal regulations on MAR in the public sector ( Art. 3.1, Law 14/2006): “Assisted reproduction techniques shall be performed only where there are reasonable chances of success, they do not pose a serious risk to the health, physical or mental health of the woman or the possible offspring, and after free and conscious acceptance of the application by the woman, who must have been earlier and duly informed of her chances of success, as well as of her risks and the conditions of that application.” The phrase “reasonable chances of success” serves as a legal threshold that limits access to fertility treatment in the public sector to women up to the age of 40. It is worth noting the disparity between the public and private sectors with respect to this issue.

## Conclusion

Success rates should be defined clearly and exclusively in terms of live birth. To ensure the validity of these rates, clinics should provide access to evidence-based data that allow independent verification. Yet none of the clinics consulted offers sources that substantiate the origin or accuracy of their data. Substantiation is not merely good practice; it is a legal requirement. The 2018 Code of Advertising and Marketing Communication of the International Chamber of Commerce (ICC) states: “Descriptions, claims or illustrations relating to verifiable facts in marketing communications should be capable of substantiation. Claims that state or imply that a particular level or type of substantiation exists must have at least the level of substantiation advertised. Substantiation should be available so that evidence can be produced without delay and upon request to the self-regulatory organisations responsible for the implementation of the Code” (Article 6).

Moreover, information on success rates should be based on the best available evidence, and clinics should offer access to underlying data for verification. These rates must also be disaggregated by age group, as generalised figures are misleading and should be avoided. Clinic websites are also commercial spaces, and claims fall under consumer protection law. In Spain, these recommendations align not only with existing laws on consumer protection and advertising (e.g., Article 7.1 of Law 3/1991; Art. 3 of Law 34/1988; ICC Code, 2018; Directive 2006/114/EC), but also with universal standards of medical ethics and informed consent. Yet none of the clinics surveyed adhere to these principles. Our findings reflect not only individual clinic practices but also the absence of standardised definitions, independent verification, and consumer-law oversight. This lack of clarity makes it difficult for patients to understand their real chances of success.

Success rate data should be monitored by external, independent authorities to prevent the publication of unverified or misleading statistics. To avoid conflicts of interest, we see no alternative but to establish a centralised public authority responsible for collecting, verifying, and managing clinic data to safeguard citizens' rights and interests. We advocate for the standardisation of success rate reporting to emphasise that these figures should not served as a basis for competition among clinics. Success rates are not universal indicators of quality, as outcomes depend heavily on each patient's individual health condition. Patients should be informed that success rates serve as contextual information rather than a definitive measure of a clinic’s competence. In our view, more robust regulation of the information provided by the fertility industry in Spain is urgently needed.

 In our view, more robust regulation of the information provided by the fertility industry in Spain is urgently needed. We believe that patients will be best served when regulators, clinics, and consumer-law authorities jointly address success-rate reporting, with genuine independence and without conflicts of interest.

## Ethics approval

This project (No. 2021.004) received ethical approval on 29/01/2021 by the Research Ethics Committee of the University of Navarra.

## Data Availability

The data underlying the results cannot be shared for confidentiality reasons. The data have been restricted due to the sensitive nature of the information provided. There is a concern that the fertility industry might exploit this information for commercial purposes. Therefore, in the Grant Agreement No. 872706 — B2-InF, signed by the Research Executive Agency of European Commission and all B2InF project partners, was established that the dissemination level of the interviews, as well as the dissemination level of the reports and thematic analyses of the interviews, is "confidential", explicitly defined as "only for members of the consortium (including the Commission Services)". (see Annex 1, p. 6/27 of the Grant Agreement nr. 872706). To request access, please email the corresponding author. Access will be granted once is confirmed that the data will be used solely for scientific research purposes and that all researchers involved in the study have no conflicts of interest. For the evaluation of the request, the Principal Investigator (PI) should submit: 1. A draft that includes, at a minimum, the hypothesis, the objectives of the research project, and the methodological framework. 2. A CV of the last 15 years for each researcher who will participate in the research and/or will have access to the data. 3. A personal statement from each researcher addressing the following points separately: In the last 15 years, neither my institution nor I, personally, have received salary, funding, grants, or personal fees from entities funded by the fertility industry (including clinics, laboratories or biopharma companies). In the last 15 years, neither my institution nor I, personally, have currently or previously collaborated with advocacy groups or civil associations related directly or indirectly to fertility issues. I have never held a position on boards of scientific, social or commercial entities funded by the fertility industry (including clinics, laboratories, or biopharma companies).
