# Supplementary material for: Analysis of online information about success rates in fertility clinics in Spain. An urgent call for action
Source: Open Res Eur. 2025 Oct 22;5:159. Originally published 2025 Jun 9. [Version 2] doi: 10.12688/openreseurope.20090.2 (PMC12640485; doi:10.12688/openreseurope.20090.2)
Supplement: Peer review history

# Reviewer report: Reviewer response for version 2

**DOI:** 10.21956/openreseurope.23336.r64194

**Published:** 2025-11-22

**Authors:** Petronela Naghi

**Affiliations:** 1 Calla—Infertility Diagnostic and Treatment Center, Oradea, Romania

The material presented raises a delicate issue within ART medical services, many clinics present various statistics on social media, which may not correspond to reality, and this is not only in Spain. However, no solution is in sight, since voluntary reporting is practiced, and coercive methods are not a solution

Is the study design appropriate and does the work have academic merit?

Yes

Is the work clearly and accurately presented and does it cite the current literature?

Yes

If applicable, is the statistical analysis and its interpretation appropriate?

Yes

Are all the source data underlying the results available to ensure full reproducibility?

Yes

Are the conclusions drawn adequately supported by the results?

Yes

Are sufficient details of methods and analysis provided to allow replication by others?

Yes

Reviewer Expertise:

I know the situation of fertility clinics because I work in the field, I am an embryologist, lab manager

I confirm that I have read this submission and believe that I have an appropriate level of expertise to confirm that it is of an acceptable scientific standard.

# Reviewer report: Reviewer response for version 1

**DOI:** 10.21956/openreseurope.21730.r58364

**Published:** 2025-08-19

**Authors:** Athanasios Papathanasiou

**Affiliations:** 1 Bourn Hall Clinic, Cambridgeshire, UK

This paper addresses an important and sensitive subject. Patients seeking fertility care are in a vulnerable position and deserve accurate, comprehensible, and verifiable information on success rates. Fertility treatment is not only expensive but also emotionally charged, and there is a clear moral and legal duty to avoid misleading claims. The UK’s HFEA provides one possible model: it verifies clinic submissions and publishes outcomes at both national and clinic level. This dual role—standard setting and independent verification—has strengthened comparability and public trust.

 The stated aim of the paper is valuable, yet not fully clear. The inclusion of only five clinics is insufficiently justified, leaving questions about selection bias and representativeness. Greater transparency around why these clinics were chosen, would strengthen the work.

 The Spanish context also complicates outcome reporting. A high proportion of patients are aged over 40 or undergo donor-egg treatment, and many are international patients who return home immediately after embryo transfer. As a result, pregnancy testing and ultrasound follow-up are often performed abroad, leaving Spanish clinics with incomplete outcome data. By contrast, clinics serving local populations (as in the UK) usually capture these outcomes themselves. This attrition limits the reliability of live-birth reporting in Spain. Since live birth rates are highly predictable once a clinical pregnancy is achieved, it may be more realistic for Spain to focus on clinical pregnancy rates as a provisional gold standard until live-birth follow-up can be made systematic.

 Definitions are central. The distinction between biochemical pregnancy, clinical pregnancy, and live birth is obvious to clinicians but not to patients, who need clear and accessible explanations. The authors are right that imprecision here undermines comparability, but it is important not to assume intentional manipulation. Often the issue is that those writing website content “think like doctors,” using technical categories without recognising how patients may misinterpret them.

 Stratification by patient age also deserves careful consideration. For own-egg cycles, age is the strongest predictor of success. Granular age bands such as <35, 35–37, and 38–40 improve precision, but when denominators are small, rates can become unstable. The HFEA approach—reporting more detailed bands at the national level but broader ones (≤37 vs ≥38) at clinic level—balances clarity with statistical reliability. Donor-egg cycles are different: outcomes are largely determined by donor age, usually ≤35. Apart from a modest fall above 45, recipient age has little predictive value. Stratifying donor-egg outcomes by recipient age therefore adds confusion rather than clarity.

 Another key issue is the denominator. Reporting per embryo transfer rather than per cycle can inflate apparent success rates, but both perspectives are valuable. Patients should understand the difference, and a simple glossary or side-by-side example would help avoid misinterpretation.

 Consumer law adds another layer. Clinic websites are commercial spaces, and claims must be truthful and not misleading. The UK experience shows that even with a regulator in place, misleading advertising persisted. Even with the Competition and Markets Authority involvement, the optimal reporting standards are still under debate. Without explicit consumer-law alignment, a new regulator in Spain might still fail to address how outcomes are marketed online.

 The tone of the article risks appearing combative towards clinics, especially larger groups. This could distract from the more important point that the problem is systemic: lack of shared definitions, lack of verification, and weak consumer-law oversight. Patients are best served when regulators, clinics, and consumer-law authorities act together. Framing the issue in this way would make the recommendations more constructive and less polarised.

 Some further points merit reflection. Publishing separate IVF and ICSI success rates can create a marketing pressure for patients to choose the technique with the higher number, even though this is a clinical decision based on indication and local practice. Suggesting that ICSI is used primarily for financial gain is not warranted. It is also important to note that ICSI carries recognised risks, meaning patient choice should not be swayed by website presentation. Similarly, claims such as “around 90% after three cycles” fall into a grey zone: they should certainly be framed carefully, but for younger patients or donor-egg cycles, cumulative outcomes can plausibly approach this magnitude. These statements are not necessarily deceptive, but they can be misinterpreted without context.

 The legal and ethical context is also important. In public systems, thresholds such as a “reasonable chance of success” are funding criteria, not prohibitions on private treatment. Patients retain autonomy, and clinics have a duty to support informed choice, not to restrict access based only on statistical probability.

 Overall, the article highlights real concerns but could be strengthened by greater methodological clarity, fuller contextualisation of Spanish practice, and more balanced framing. A stronger case would come from a larger and more representative sample, clearer justification for methodological choices, and explicit engagement with both consumer law and the realities of international medical tourism.

 Despite its limitations, the paper raises critical issues and should be viewed as a stepping stone towards a more transparent, patient-protective framework in Spain. The best way forward will likely involve a national authority that combines standard-setting, independent verification, and alignment with consumer-law enforcement. This would improve clarity and comparability for patients while supporting clinics in providing trustworthy information.

Is the study design appropriate and does the work have academic merit?

Yes

Is the work clearly and accurately presented and does it cite the current literature?

Partly

If applicable, is the statistical analysis and its interpretation appropriate?

Not applicable

Are all the source data underlying the results available to ensure full reproducibility?

Partly

Are the conclusions drawn adequately supported by the results?

Partly

Are sufficient details of methods and analysis provided to allow replication by others?

Partly

Reviewer Expertise:

I have been running a group of UK fertility clinics and have been working closely with UK regulators and consumer law authorities. My areas of research include: Poor Ovarian Response (POR) during IVF, Emotional Aspects of IVF, Improving access to IVF treatment.

I confirm that I have read this submission and believe that I have an appropriate level of expertise to confirm that it is of an acceptable scientific standard, however I have significant reservations, as outlined above.

# Response

**Published:** 2025-10-05

**Authors:** Anna De Bayas Sanchez

**Affiliations:** Not Applicable, Czech Republic

We would like to thank you sincerely for your careful reading of our manuscript and for your thoughtful and constructive comments. Your observations have been extremely valuable in helping us to improve the clarity, quality, and overall rigour of our work. Below, we provide a point-by-point response to each of your numbered comments. For each one, we explain the changes made to the manuscript and where they can be found in the revised version. ---------

**This paper addresses an important and sensitive subject. Patients seeking fertility care are in a vulnerable position and deserve accurate, comprehensible, and verifiable information on success rates. Fertility treatment is not only expensive but also emotionally charged, and there is a clear moral and legal duty to avoid misleading claims. The UK**’ **s HFEA provides one possible model: it verifies clinic submissions and publishes outcomes at both national and clinic level. This dual role—standard setting and independent verification—has strengthened comparability and public trust.**

 Thank you for your comment. We are familiar with the UK’s HFEA model and recognise its value. However, the aim of this article is not to propose a specific regulatory model, but rather to analyse the information as it is presented from the patient’s perspective. In other words, our objective is to examine the clarity and accuracy of the information provided by private companies in Spain. Our findings lead us to emphasise that presenting clear and truthful success-rate information is not only a legal obligation but also an ethical duty in reproductive healthcare. Moreover, given that current legislation on information is not being effectively enforced, we consider that urgent action by regulatory authorities is required. The form that such action should take, however, lies beyond the scope of this article.

**The stated aim of the paper is valuable, yet not fully clear. The inclusion of only five clinics is insufficiently justified, leaving questions about selection bias and representativeness. Greater transparency around why these clinics were chosen, would strengthen the work.** Thank you for your helpful suggestion. We have revised the text to clarify why we focused on five clinics and to acknowledge the limitations of this approach. We added the following paragraph: “We conducted a systematic analysis of success-rate information published on the websites of Spain’s five MAR clinics with the strongest online presence as of November 2023.”  “Our focus was on the patient perspective: patients typically rely on online searches rather than scientific registries when seeking fertility care. Therefore, clinics most visible on Google are the most influential in shaping patient decisions. While this approach does not capture the full universe of Spanish clinics, it provides relevant insights into the information that patients are most likely to encounter.”

**The Spanish context also complicates outcome reporting. A high proportion of patients are aged over 40 or undergo donor-egg treatment, and many are international patients who return home immediately after embryo transfer. As a result, pregnancy testing and ultrasound follow-up are often performed abroad, leaving Spanish clinics with incomplete outcome data. By contrast, clinics serving local populations (as in the UK) usually capture these outcomes themselves. This attrition limits the reliability of live-birth reporting in Spain. Since live birth rates are highly predictable once a clinical pregnancy is achieved, it may be more realistic for Spain to focus on clinical pregnancy rates as a provisional gold standard until live-birth follow-up can be made systematic.** Thank you very much for this valuable comment. While some Spanish clinics have a high proportion of international patients, these represent only about 10–15% nationwide (11% according to the latest SEF report). The number of domestic patients alone is larger than in most European countries, providing a robust dataset for estimating live-birth success rates. From a patients’ rights perspective, the challenge of follow-up cannot exempt clinics from reporting the live-birth outcome, which is both a legal requirement and the central figure patients need for informed decision-making. In any case, we are grateful for your valuable suggestion, and we have incorporated into the article, following your orientation, the following paragraph:

  “Although some fertility clinics in Spain may have a high proportion of international patients, they represent only about 10% of all treatment cycles nationwide, according to the latest reports from the Spanish Fertility Society (SEF). The number of domestic patients alone is higher than that reported by most other countries, providing a sufficiently large and robust dataset to estimate live-birth success rates with confidence.”

**Definitions are central. The distinction between biochemical pregnancy, clinical pregnancy, and live birth is obvious to clinicians but not to patients, who need clear and accessible explanations. The authors are right that imprecision here undermines comparability, but it is important not to assume intentional manipulation. Often the issue is that those writing website content **“ **think like doctors,” using technical categories without recognising how patients may misinterpret them.**

 Thank you very much for this helpful comment. We fully agree that it is important not to assume intentional manipulation. We have carefully reviewed the manuscript with this in mind, and we believe that in its current form there are no statements that imply intentionality. To clarify this further, we have added the following paragraph: Our findings reflect not only individual clinic practices but also the absence of standardised definitions, independent verification, and consumer-law oversight. This lack of clarity makes it difficult for patients to understand their real chances of success.

**Stratification by patient age also deserves careful consideration. For own-egg cycles, age is the strongest predictor of success. Granular age bands such as <35, 35–37, and 38–40 improve precision, but when denominators are small, rates can become unstable. The HFEA approach—reporting more detailed bands at the national level but broader ones (≤37 vs ≥38) at clinic level—balances clarity with statistical reliability. Donor-egg cycles are different: outcomes are largely determined by donor age, usually ≤35. Apart from a modest fall above 45, recipient age has little predictive value. Stratifying donor-egg outcomes by recipient age therefore adds confusion rather than clarity.** Thank you very much for this valuable comment. We have added a paragraph on this important issue to bring further clarity: Although donor age is the principal determinant of success in donor-egg IVF, recent evidence shows that recipient age also significantly influences outcomes, with implantation failure and pregnancy loss risks increasing progressively after the age of 40 (Sebastian-Leon et al., 2025). This indicates that both own-egg and donor-egg cycles benefit from more precise age stratification than is currently provided in Spain.

**Another key issue is the denominator. Reporting per embryo transfer rather than per cycle can inflate apparent success rates, but both perspectives are valuable. Patients should understand the difference, and a simple glossary or side-by-side example would help avoid misinterpretation.** Thank you very much for this helpful comment. We have addressed this point by adding the following clarification: “Another dimension not addressed on clinic websites is whether success rates are reported per initiated cycle, per egg retrieval, or per embryo transfer. These yield different figures, and without clarification patients may misinterpret the likelihood of success. Providing both perspectives with clear explanations would enhance transparency”.

**Consumer law adds another layer. Clinic websites are commercial spaces, and claims must be truthful and not misleading. The UK experience shows that even with a regulator in place, misleading advertising persisted. Even with the Competition and Markets Authority involvement, the optimal reporting standards are still under debate. Without explicit consumer-law alignment, a new regulator in Spain might still fail to address how outcomes are marketed online.** Thank you very much for this very interesting reflection. We fully recognise the value of the UK experience, as well as those of Australia or the United States. However, as stated before, our goal in this article is not to undertake cross-country comparisons, but rather to analyse the information available to patients in Spain. We hope that future research will explore these international perspectives more fully.

**The tone of the article risks appearing combative towards clinics, especially larger groups. This could distract from the more important point that the problem is systemic: lack of shared definitions, lack of verification, and weak consumer-law oversight. Patients are best served when regulators, clinics, and consumer-law authorities act together. Framing the issue in this way would make the recommendations more constructive and less polarised.** We sincerely appreciate your observation regarding the tone of the article. We acknowledge that, at first reading, it may appear somewhat combative towards clinics. However, our intention was not to single out specific institutions, but rather to report factual issues and patterns that are observable in the field. We also agree that framing the matter as a systemic problem is essential. One of our main concerns is precisely the risk of polarization between clinics and a general public that often lacks reliable information. We therefore agree that collaboration between regulators, clinics, and consumer-law authorities is necessary. At the same time, we would underline that patients will be best served when such cooperation takes place under conditions of genuine independence and free from conflicts of interest. To address your suggestion, we have incorporated clarifying sentences such as: “Our findings reflect not only individual clinic practices but also the absence of standardised definitions, independent verification, and consumer-law oversight.” Finally, we conclude with: “We believe that patients will be best served when regulators, clinics, and consumer-law authorities jointly address success-rate reporting, with genuine independence and without conflicts of interest.”

**Some further points merit reflection. Publishing separate IVF and ICSI success rates can create a marketing pressure for patients to choose the technique with the higher number, even though this is a clinical decision based on indication and local practice. Suggesting that ICSI is used primarily for financial gain is not warranted. It is also important to note that ICSI carries recognised risks, meaning patient choice should not be swayed by website presentation. Similarly, claims such as **“ **around 90% after three cycles” fall into a grey zone: they should certainly be framed carefully, but for younger patients or donor-egg cycles, cumulative outcomes can plausibly approach this magnitude. These statements are not necessarily deceptive, but they can be misinterpreted without context.** Thank you very much for this thoughtful comment. We agree that it is important to present success rates in a way that avoids creating marketing pressure or misinterpretation. To address this, we have added the following clarification: “Given ICSI’s widespread use in Spain, greater transparency would allow patients to understand when IVF and ICSI are clinically indicated.”

**The legal and ethical context is also important. In public systems, thresholds such as a **“ **reasonable chance of success” are funding criteria, not prohibitions on private treatment. Patients retain autonomy, and clinics have a duty to support informed choice, not to restrict access based only on statistical probability.** We appreciate your point regarding the legal and ethical context. To clarify, our article does question the effective autonomy of patients, but not in the sense of restricting their choices. Rather, we argue that genuine autonomy is not possible when patients lack accurate, transparent, and comprehensible information about success rates. We are not attempting to define what percentage of success should be considered “reasonable,” nor are we suggesting that access should be limited on this basis. Our focus is solely on the absence of transparency and clarity in reporting, which undermines the conditions under which informed and autonomous decision-making can truly take place. --------- We are very grateful for your contribution to strengthening our submission. Yours sincerely,

  Anna de Bayas Sanchez

# Reviewer report: Reviewer response for version 1

**DOI:** 10.21956/openreseurope.21730.r55326

**Published:** 2025-07-14

**Authors:** Karin Hammarberg

**Affiliations:** 1 Monash University, Clayton, Victoria, Australia

Thank you for asking me to review this paper which reports findings from an analysis of content relating to success rates on fertility clinic websites in Spain. This is a very important topic as there is increasing understanding that the commercial interests in the fertility industry may impact negatively the quality of information for patients about what is possible with IVF. I have the following questions and comments.

1. Title: I suggest removing ‘public health’ and just having ‘action’ as in my view this has nothing to do with public health.
2. Abstract: What is meant by ‘five most representative MAR clinics”? Representative in what way? Should that be largest?
3. Abstract: Similarly, it’s unclear what you mean by ‘legal recommendations’, might be better to just have ‘recommendations’.
4. Last paragraph before methods: Here you give away the answer to the question you are posing. I would take out reference to how clinics present success rates as that is your research question. Also, the last sentence states that this is the first study on this topic. This is incorrect, there are others. It might be the first analysis of content on Spanish fertility clinic websites. 
5. Methods: Was there a reason for not involving patients or the public in the study design?? As this mainly concerns patients, their input would have been valuable.
6. Methods: You say the same thing twice here: ‘To identify **the most frequently consulted MAR clinics in Spain**, we conducted internet searches using the five most common Google search keywords related to fertility treatment: ‘fertilisation’, ‘insemination‘, ‘infertility’, ‘pregnancy IVF’ (in vitro fertilisation), and ‘ICSI’ (intracytoplasmic sperm injection). We chose this selection method **to identify the most frequently visited and consulted clinics in Spain**, ensuring that their online information is the most visible to the general public.’ Please rephrase.
7. You mention that one of the things you looked for was ‘certification and auditing by external bodies’. You should add some information in the introduction about how ART is regulated in Spain. Are clinics obliged to be certified to operate and if so, who does this? Also, are clinics mandated to report their outcomes (number of cycles, pregnancy and live birth etc) to any central data collector and are such data published??
8. In the results section I suggest you organise the data you report under subheadings that match the data you say you collected in the methods section. This would make it easier for readers to follow.
9. Results: You say ‘In particular, we analysed whether the success rate is defined as live birth, clinical pregnancy, or chemical pregnancy, and whether these criteria are explicitly stated and explained.’ I think you should also mention what denominator clinics use for their success rate reporting. Often clinics report outcome data per embryo transfer which does not account for the people who don’t reach the embryo transfer stage.
10. The first paragraph under the heading ‘Online information on success rate and age’ is not part of your results, would be better placed in the introduction.
11. The last sentence in the first column on page 5 says that clinics did not report IVF and ICSI outcomes separately. In some clinics ICSI is used in a high proportion of patients, including in cases where there is no male factor (against the existing evidence that this does not improve their chance of a baby). It would be helpful to know what proportion of ICSI cycles in the clinics you included.
12. This would be better placed in the discussion as it is not part of your results: ‘In fact, based on the data provided by the French government, the success rate of ICSI is only slightly higher than that of IVF, and not always. Studies indicate that when the cause of infertility is not of paternal origin, the success rates of IVF are better than those of ICSI, although it is debatable which technique is more beneficial in other cases (Bantel-Finet et al., 2022). In light of this evidence, and given the different costs associated with using one or the other, information about success rates should enable patients to take these factors into consideration.’
13. In that same paragraph you say that clinics were ‘interviewed’, wasn’t this a content review??
14. On page 6 this would be better placed in the discussion in my view: ‘Such statements violate Spanish and European consumer protection laws on truthful advertising, which prohibit any advertising that, through false or misleading information—or the omission of essential details—leads consumers to make misinformed decisions.’
15. This is self-evident and does not in my view need stating: ‘The primary expectation of people seeking fertility treatment is to become the parent of a healthy child—the main reason they seek the services of a clinic.’ The sentence that follows mentions ‘requirement’ but it is not clear what the requirement is.
16. The first part on page 7 repeats the results, this should be avoided.
17. The sentence that follows starts with ‘Given this expectation…’ What expectation?
18. The last sentence in that paragraph states that patients receive a ‘success rate defined exclusively in terms of live birth’. It may be better to say that they receive an estimate of their chance of a live birth based on the data they enter.
19. The conclusion is very long and the first sentence can be omitted as it is self-evident.

Is the study design appropriate and does the work have academic merit?

Yes

Is the work clearly and accurately presented and does it cite the current literature?

Partly

If applicable, is the statistical analysis and its interpretation appropriate?

Not applicable

Are all the source data underlying the results available to ensure full reproducibility?

Partly

Are the conclusions drawn adequately supported by the results?

Yes

Are sufficient details of methods and analysis provided to allow replication by others?

Partly

Reviewer Expertise:

PhD and co-ordinator of IVF programs for 20 year.

I confirm that I have read this submission and believe that I have an appropriate level of expertise to confirm that it is of an acceptable scientific standard, however I have significant reservations, as outlined above.

# Response

**Published:** 2025-10-05

**Authors:** Anna De Bayas Sanchez

**Affiliations:** Not Applicable, Czech Republic

We would like to thank you sincerely for your careful reading of our manuscript and for your thoughtful and constructive comments. Your observations have been extremely valuable in helping us to improve the clarity, quality, and overall rigour of our work. Below, we provide a point-by-point response to each of your numbered comments. For each one, we explain the changes made to the manuscript and where they can be found in the revised version. --------

 1. Title: I suggest removing ‘public health’ and just having ‘action’ as in my view this has nothing to do with public health. 

**Response: **Thank you for your suggestion. We have removed the reference to “public health” from the title, as recommended, and now refer simply to “action.” We agree that this change makes the title more accurate and focused.

 2. Abstract: What is meant by ‘five most representative MAR clinics”? Representative in what way? Should that be largest?  

**Response: **We agree that “representative” may imply broader coverage than intended. We have revised the text to clarify that our selection was based on online visibility and patient-facing influence, rather than clinic size or national representativeness.

 3. Abstract: Similarly, it’s unclear what you mean by ‘legal recommendations’, might be better to just have ‘recommendations.****

**Response: **Thank you for this helpful suggestion. We agree that the term “legal recommendations” could be misleading or overly narrow. Our intention was to propose recommendations aimed at improving transparency, consumer protection, and advertising standards in the context of fertility treatment communication. To avoid confusion, we have revised the wording to “recommendations aimed at improving consumer protection and regulating advertising practices” in the Results section.

 4. Last paragraph before methods: Here you give away the answer to the question you are posing. I would take out reference to how clinics present success rates as that is your research question. Also, the last sentence states that this is the first study on this topic. This is incorrect, there are others. It might be the first analysis of content on Spanish fertility clinic websites.

**Response: **Thank you very much for this helpful observation. We have revised the final paragraph of the Introduction to avoid anticipating the findings of the study and have removed the reference to how clinics present success rates. We have also rephrased the final sentence to clarify that, to our knowledge, this is the first systematic content analysis of Spanish fertility clinic websites focused specifically on success rate information.

 5. Methods: Was there a reason for not involving patients or the public in the study design? As this mainly concerns patients, their input would have been valuable. 

**Response: **In fact, we did involve the largest patient association, “Fertility Europe”, which is part of the B2-InF consortium (although not of the scientific team), in a meeting where we discussed the checklist of data to be collected from clinic websites (of which success rates were only one of the aspects analysed). Thank you for your comment and for helping us to recognize the added value of that preliminary consultation. We have added this information to the Methods

 6. Methods: You say the same thing twice here: ‘To identify the most frequently consulted MAR clinics in Spain, we conducted internet searches using the five most common Google search keywords related to fertility treatment: ‘fertilisation’, ‘insemination‘, ‘infertility’, ‘pregnancy IVF’ (in vitro fertilisation), and ‘ICSI’ (intracytoplasmic sperm injection). We chose this selection method to identify the most frequently visited and consulted clinics in Spain, ensuring that their online information is the most visible to the general public.’ 

**Response: ** Thank you for pointing this out. We have revised the paragraph to eliminate redundancy and improve clarity. The revised text now avoids repetition while clearly explaining the rationale and method for selecting the clinics based on their online visibility.

 7. You mention that one of the things you looked for was ‘certification and auditing by external bodies’. You should add some information in the introduction about how ART is regulated in Spain. Are clinics obliged to be certified to operate and if so, who does this? Also, are clinics mandated to report their outcomes (number of cycles, pregnancy and live birth etc) to any central data collector and are such data published?

**Response: **Thank you for this helpful observation. We have now added a paragraph to the introduction explaining the regulatory framework for ART clinics in Spain. Specifically, we clarify that clinics must be authorised by the regional health authorities, in accordance with national law, and that they are required to report their treatment activity and outcomes to the Spanish Fertility Society (SEF). The SEF publishes aggregated national data annually, although clinic-level data publication remains voluntary. The revised introduction provides this context, which helps to better frame our analysis of the lack of transparency and certification in the reporting of success rates.

 8. In the results section I suggest you organise the data you report under subheadings that match the data you say you collected in the methods section. This would make it easier for readers to follow. 

**Response: **Thank you very much for this helpful observation. We have revised the order of the analysis as described in the Methods section so that it now matches the structure and subheadings used in the Results section. We agree that this improves the clarity and readability of the article.

 9. Results: You say ‘In particular, we analysed whether the success rate is defined as live birth, clinical pregnancy, or chemical pregnancy, and whether these criteria are explicitly stated and explained.’ I think you should also mention what denominator clinics use for their success rate reporting. Often clinics report outcome data per embryo transfer which does not account for the people who don’t reach the embryo transfer stage.

**Response: **Thank you for your thoughtful comment. Indeed, we agree that the choice of denominator is crucial for interpreting success rates. In the case of the clinics analysed, all of them report success rates in terms of cumulative cycle outcomes. We have clarified this point in the Results section to make it explicit.****

 10. The first paragraph under the heading ‘Online information on success rate and age’ is not part of your results, would be better placed in the introduction.****

**Response: **Thank you very much for your comment. We agree that the paragraph in question did not report results and therefore was not appropriate in the Results section. We have now removed it from that section and integrated it into the Introduction, where it fits more naturally and contributes to the contextual justification of our analysis. We believe this change improves the structure and clarity of the manuscript.

 11. The last sentence in the first column on page 5 says that clinics did not report IVF and ICSI outcomes separately. In some clinics ICSI is used in a high proportion of patients, including in cases where there is no male factor (against the existing evidence that this does not improve their chance of a baby). It would be helpful to know what proportion of ICSI cycles in the clinics you included. 

**Response: **Thank you for your thoughtful comment. We contacted the Spanish Fertility Society (SEF) to request access to clinic-specific data regarding the proportion of ICSI cycles. However, we were informed that "access to specific data from the Activity Registry – SEF Registry is not contemplated." Therefore, we have no means of accessing this information at the individual clinic level. Nonetheless, national-level data are available. According to the 2022 SEF Registry, ICSI accounted for approximately 88.2% of initiated cycles in Spain. We have now included this national statistic in the manuscript, as you suggested, to provide contextual information about the widespread use of ICSI in Spain.

 12. This would be better placed in the discussion as it is not part of your results: ‘In fact, based on the data provided by the French government, the success rate of ICSI is only slightly higher than that of IVF, and not always. Studies indicate that when the cause of infertility is not of paternal origin, the success rates of IVF are better than those of ICSI, although it is debatable which technique is more beneficial in other cases (Bantel-Finet et al., 2022). In light of this evidence and given the different costs associated with using one or the other, information about success rates should enable patients to take these factors into consideration.’

**Response: **Thank you for your comment. We agree that the paragraph you mentioned does not correspond to the results of our empirical analysis. We have therefore removed it from the Results section and integrated it into the Discussion, where it contributes more appropriately to the interpretation of the findings and their implications for patient decision-making.

 13. In that same paragraph you say that clinics were ‘interviewed’, wasn’t this a content review?

**Response: **Thank you for catching this miswording. You are absolutely right - the clinics were not interviewed. The data were obtained through a systematic content review of their websites. We have corrected the sentence to reflect this accurately.****

 14. On page 6 this would be better placed in the discussion in my view: ‘Such statements violate Spanish and European consumer protection laws on truthful advertising, which prohibit any advertising that, through false or misleading information—or the omission of essential details—leads consumers to make misinformed decisions.”

**Response: **Thank you for this suggestion. We have removed the sentence from the Results section and placed it in the Discussion, where it is more appropriately situated within the analysis of legal compliance and consumer protection.

 15. This is self-evident and does not in my view need stating: ‘The primary expectation of people seeking fertility treatment is to become the parent of a healthy child—the main reason they seek the services of a clinic.’ The sentence that follows mentions ‘requirement’ but it is not clear what the requirement is. 

**Response: **Thank you for your helpful comment. You are right that the original formulation was unclear and could be seen as self-evident. We have revised the paragraph, accordingly, removing the sentence about patients' expectations and clarifying the reference to live birth as the most relevant outcome. We believe this improves both the clarity and precision of the argument.

 16. The first part on page 7 repeats the results, this should be avoided.****

**Response: **Thank you for pointing this out. We have revised the first part of page 7 to avoid repeating the results already presented. Instead of reiterating the data, we now provide a critical interpretation and highlight the ethical and legal implications of the lack of transparency in reporting success rates. This change strengthens the discussion by focusing on analysis rather than description.

 17. The sentence that follows starts with ‘Given this expectation…’ What expectation?**** **Response: **Thank you for your comment. We agree that the paragraph in question repeated information already presented in the Results section. We have removed it and revised the Discussion section, accordingly, focusing on the ethical, legal, and policy implications of our findings rather than restating the data.

 18. The last sentence in that paragraph states that patients receive a ‘success rate defined exclusively in terms of live birth’. It may be better to say that they receive an estimate of their chance of a live birth based on the data they enter.  

**Response: **Thank you for this helpful clarification. We agree that the original wording overstated the level of precision and determinism. We have revised the sentence to reflect that patients receive an estimate of their chance of live birth, based on the clinical data they provide and national datasets.****

 19. The conclusion is very long, and the first sentence can be omitted as it is self-evident.****

**Response: **Thank you for your helpful suggestion. We have removed the first sentence of the conclusion and slightly reduced the overall length of the paragraph to improve clarity and conciseness, while retaining all key ideas. We appreciate your feedback. -------- We are very grateful for your contribution to strengthening our submission. Yours sincerely,

  Anna de Bayas Sanchez
